# *In silico* analysis of a SLC6A4 G100V mutation in lung cancers

**DOI:** 10.17912/micropub.biology.000645

**Published:** 2022-09-27

**Authors:** Amrit L Pappula, Louis N Gibson, Renee A Bouley, Ruben C Petreaca

**Affiliations:** 1 The Ohio State University

## Abstract

SLC6A4 is a serotonin re-uptake transporter which has been a target for anti-depressant therapies but recently some mutations have been described in cancer cells. Here, we characterize mutations in SLC6A4 that appear in cancer cells. We employed several validated computational and artificial intelligence algorithms to characterize the mutations. We identified a previously uncharacterized G100V mutation in lung cancers. *In sillico* structural analysis reveals that this mutation may affect SLC6A4 ligand binding and subsequently its function. We also identified several other mutations that may affect the structure of the protein. This preliminary analysis highlights the role of SLC6A4 in human cancers.

**Figure 1. Identification of a G100V SLC6A4 mutation in human lung cancers.**
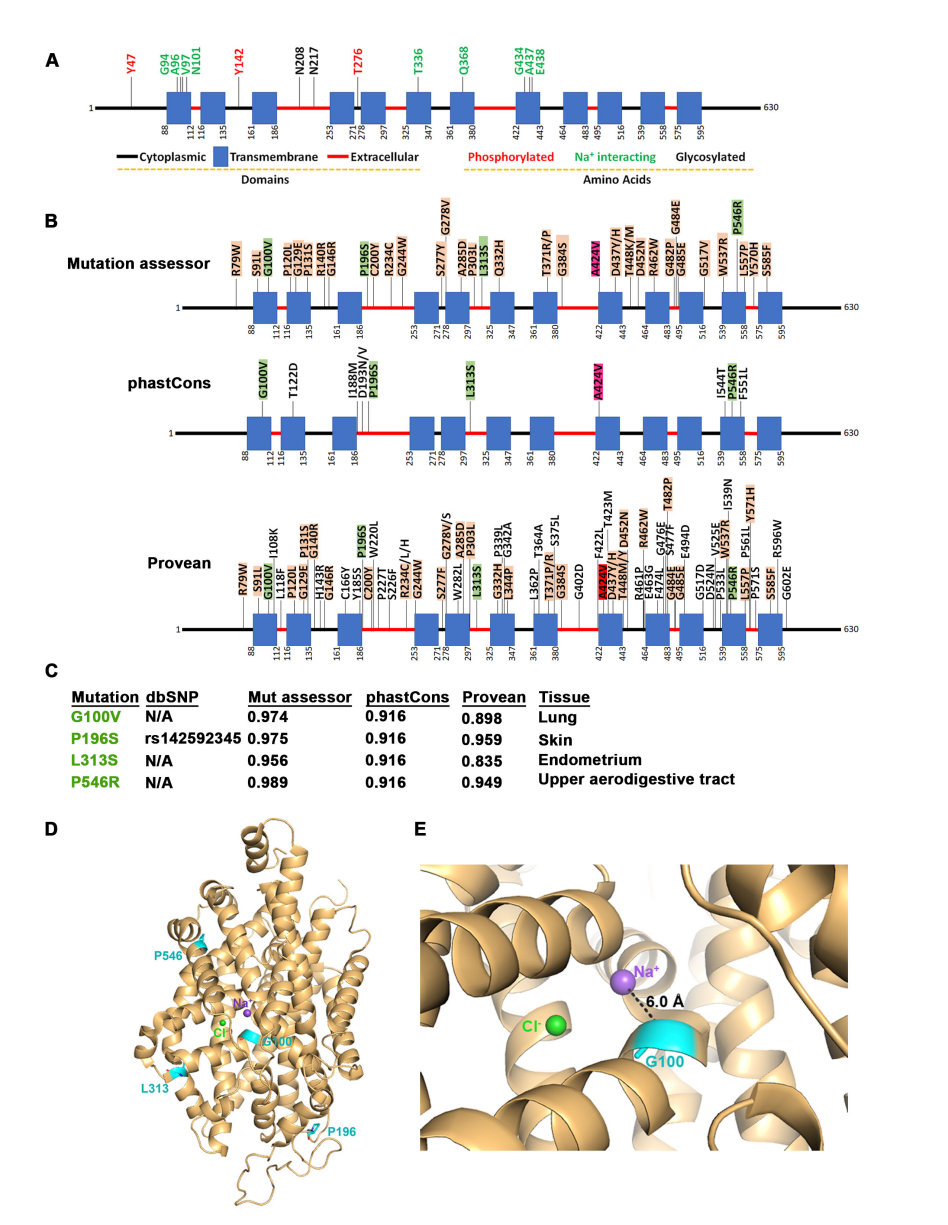
A. SLC6A4 protein domain architecture and modified residues. Diagram based on information from UniProt (UniProtKB - P31645). B. Determination of mutation pathogenicity and/or driver status. A. Distribution of mutations deemed significant by the three algorithms employed. Please see Methods for description. C. Four mutations that were identified as significant by all three algorithms. Indicated are algorithm scores and corresponding dbSNP identifier if available. D. Location of the four identified mutations on an X-ray structure of SLC6A4. Mutated residues are highlighted in cyan and shown as sticks on a published crystal structure of SLC6A4 (PDB ID: 5I6X). The bound sodium and chloride ions are shown as spheres in purple and green respectively. E. Zoomed in view of Gly100 showing measured distance between the Ca atom and the sodium.

## Description

SLC6A4 is a serotonin re-uptake transporter which functions to recycle serotonin from the synaptic cleft and is known by several names including 5-HTT (Hamon et al., 1990). X-ray and Cryo-EM analysis as well as physiological experiments show that its function is sodium dependent (Coleman et al., 2016; Coleman et al., 2019; Szollosi and Stockner, 2022). The transporter is characterized by 12 transmembrane domains (Fig.1A) (Chen et al., 1998; Nelson, 1998). Both the domains and the non-transmembrane loops, particularly the extracellular loops, are involved in the serotonin translocation mechanism (Barker et al., 1999; Chen and Rudnick, 2000; Fenollar-Ferrer et al., 2014; Just et al., 2004; Sato et al., 2004; Smicun et al., 1999; Stephan et al., 1997). Phosphorylation of residues Y47, Y142 and T276 increases transporter activity (Annamalai et al., 2012; Zhang et al., 2007) while glycosylation at N208 and N217 increases its membrane stability (Coleman et al., 2016; Tate and Blakely, 1994). Several sodium binding residues have also been identified (Coleman et al., 2016).

The transporter has been a target for anti-depressants (Stein et al., 2021). Among the genetic variations, an HTTLPR polymorphism has been identified in the promoter region (Heils et al., 1995; Nakamura et al., 2000) that gives rise to two alleles (L and S) characterized by 16 and 14 HTTLPR repeats, respectively (Lesch et al., 1996). The L allele has been linked to obsessive compulsive disorders (Hu et al., 2006) but carriers of at least one L allele were more likely to respond to anti-depressant therapy (e.g., better prognosis for LL and LS compared to SS) (Stein et al., 2021; Wilkie et al., 2009). The S allele is associated with depression (Fratelli et al., 2020; Palma-Gudiel and Fananas, 2017) and suicide, particularly repeated suicide attempts (Antypa et al., 2013; De Berardis et al., 2021; Mirkovic et al., 2016). The distribution of the S and L alleles are dissimilar among world populations (Murphy et al., 2013). Other alleles have been correlated with coffee consumption (Coffee et al., 2015), social adversity (Surtees et al., 2006), alcohol consumption (Munafo et al., 2005), smoking (Liu et al., 2005), as well as other factors (Homberg and Lesch, 2011).

SLC6A4 is expressed primarily in the lung and the intestines (Fagerberg et al., 2014). Recent evidence has shown that the transporter is over-expressed in non-small cell lung cancer and this correlates with poor prognosis because it activates the C-Myc oncogene (Tu et al., 2022). Inhibition of SLC6A4 has also been associated with decreased tumor proliferation in colorectal cancers (Fang et al., 2012; Ye et al., 2021). Various other SLC6A4 polymorphic alleles with differential effects on tumor progression have been described in the literature (Chamba et al., 2010; Hallett et al., 2016; Ouyang et al., 2018; Phi van et al., 2015; Savas et al., 2012; Serafeim et al., 2002; Serafeim et al., 2003; Yoshimura et al., 2003; Zharinov et al., 2021).

Here, we queried the Catalogue of Somatic Mutations in Cancers (COSMIC) (Tate et al., 2019) to categorize SLC6A4 mutations reported in human cancers. Gene expression and copy number variants were excluded from this analysis. There were 307 non-coding and 353 coding mutations identified in all reported cancers. When we cataloged the coding mutations by missense, non-sense/frameshift, and synonymous/silent, we found that they distribute throughout the entire region of the protein. We identified some mutations that occur at higher frequency. Two of the mutations (AS419= and G25R) are known SNPs classified on ClinVar as being of “uncertain significance”. An A419V substitution as well as a frameshift/truncation mutation (G25*) was also identified. Neither the A419V nor the G25* mutations have been reported on ClinVar. Four other “hotspots” at R144, V457, H75R and R234 were identified. Notably, these mutations are not restricted to one cancer type indicating that there is nothing significant about the physiology of the tissue where they occur.

To understand which mutations are likely to affect the protein function, we employed several published algorithms for classification (see methods section). We found that the statistically relevant mutations identified by the three algorithms distribute throughout the entire protein sequence (Fig.1B). Several mutations were identified by more than one algorithm which indicates that these mutations are more likely to affect the gene function. We identified four mutations that were predicted by all three algorithms ( *Mutation assessor* , *phastCons* and *Provean* ) to be significant (Fig.1C).

To better understand the significance of these mutations we used a previously published crystal structure of the human serotonin transporter (PDB ID: 5I6X) (Coleman et al., 2016). This structure also had a ligand bound (paroxetine) and a sodium ion, which would allow us to determine if mutations could disrupt ligand or sodium or binding. Of the four mutated residues, only G100 was located within the ligand binding site of the protein (Fig.1D). Indeed, it is located only 6.0 Å from the bound sodium ion and the valine substitution could interfere with its binding (Fig.1E). Two of the mutated residues were proline residues, which when mutated could affect the secondary structure of the protein.

Although SLC6A4 mutations have been previously linked with various psychological and neurological disorders, recent studies have implicated this gene in cancer. Here we identified a G100V point mutations that is likely to affect the function of this gene. Our study, although preliminary, highlights the importance of SLC6A4 mutations in cancers and opens the door for further investigations of the role of this gene in cellular transformation and immortalization.

## Methods

COSMIC deposits data from various sources including The Cancer Genome Atlas (TCGA) and independent studies. An Excel file with SLC6A4 mutation data was downloaded from COSMIC V95. The file contains both coding and non-coding mutations (5’, 3’ UTR, and intronic).

Three different algorithms have been used to determine impact of mutations. *Mutation assessor* ranks mutations by combining various factors including frequency, the gene’s role in cancer, and protein function impact (Reva et al., 2007; Reva et al., 2011). The score varies between 0 and 1 with mutations above 0.95 considered to have significant high functional impact. *phastCons* identifies evolutionary conserved residues with the scores representing probability of negative selection and ranging between 0 and 1 (Siepel et al., 2005). A high level of evolutionary conservation suggests that the residue is important for the function of the protein, and we wanted to know whether mutations occur in these residues. *Provean* predicts how mutations affect protein function (Choi and Chan, 2015; Choi et al., 2012) with scores above 0.7 considered damaging. For all analyses, we used OpenCRAVAT tool which houses all the above-mentioned algorithms (https://opencravat.org/index.html)(Pagel et al., 2020).

All figures were made in Photoshop. Figures 1D and 1E was generated using PyMOL.

## Reagents

N/A
